# TGF-β-Neutralizing Antibody 1D11 Enhances Cytarabine-Induced Apoptosis in AML Cells in the Bone Marrow Microenvironment

**DOI:** 10.1371/journal.pone.0062785

**Published:** 2013-06-27

**Authors:** Yoko Tabe, Yue Xi Shi, Zhihong Zeng, Linhua Jin, Masato Shikami, Yasuhito Hatanaka, Takashi Miida, Frank J. Hsu, Michael Andreeff, Marina Konopleva

**Affiliations:** 1 Department of Leukemia, The University of Texas MD Anderson Cancer Center, Houston, Texas, United States of America; 2 Department of Clinical Laboratory Medicine, Juntendo University School of Medicine, Tokyo, Japan; 3 Department of Hematology, Aichi Medical University, Aichi, Japan; 4 Genzyme Corporation, Cambridge, Massachusetts, United States of America; University of Medicine and Dentistry of New Jersey, United States of America

## Abstract

Hypoxia and interactions with bone marrow (BM) stromal cells have emerged as essential components of the leukemic BM microenvironment in promoting leukemia cell survival and chemoresistance. High levels of transforming growth factor beta 1 (TGFβ1) produced by BM stromal cells in the BM niche regulate cell proliferation, survival, and apoptosis, depending on the cellular context. Exogenous TGFβ1 induced accumulation of acute myeloid leukemia (AML) cells in a quiescent G_0_ state, which was further facilitated by the co-culture with BM-derived mesenchymal stem cells (MSCs). In turn, TGFβ-neutralizing antibody 1D11 abrogated rhTGFβ1 induced cell cycle arrest. Blocking TGFβ with 1D11 further enhanced cytarabine (Ara-C)–induced apoptosis of AML cells in hypoxic and in normoxic conditions. Additional constituents of BM niche, the stroma-secreted chemokine CXCL12 and its receptor CXCR4 play crucial roles in cell migration and stroma/leukemia cell interactions. Treatment with 1D11 combined with CXCR4 antagonist plerixafor and Ara-C decreased leukemia burden and prolonged survival in an *in vivo* leukemia model. These results indicate that blockade of TGFβ by 1D11 and abrogation of CXCL12/CXCR4 signaling may enhance the efficacy of chemotherapy against AML cells in the hypoxic BM microenvironment.

## Introduction

Hypoxia and interactions with bone marrow (BM) stromal cells have emerged as essential components of the leukemic BM microenvironment in promoting leukemia cell survival and chemoresistance [1]. BM stromal cells in the BM niche produce several secreted growth factors, including high levels of transforming growth factor beta (TGF-β) [2] which is also released from the bone by osteoclasts [3]. The multifunctional TGF-β regulates cell proliferation, survival, and apoptosis, depending on the cellular context [4], [5]. The three major mammalian TGF-β isoforms are TGF-β1, TGF-β2, and TGF-β3; TGF-β1 is the most abundant, universally expressed isoform [6]. Following extracellular activation, TGF-β binds to the type II TGF-β receptor (TβR-II), which then recruits and activates the type I receptor (TβR-I/Alk-5) [7]. The activated TβR-I/Alk-5 transduces signals into the cytoplasm through phosphorylation of Smads, thus activating Smad2 and/or Smad3, which form complexes with common mediator Smad4. These activated Smad complexes accumulate in the nucleus, where they participate in transcriptional activation of target genes [6], [8].

Exogenous TGF-β1 has been demonstrated to directly arrest growth [9], [10] and prevent serum deprivation–induced apoptosis in leukemic cells [11], [12]. Further, TGF-β1 was shown to stimulate secretion of interleukin (IL)-6 and vascular endothelial growth factor by BM stromal cells which in turn promotes survival of myeloma cells [13]. The TGF-β–Smad pathway is also known to induce production of extracellular matrix component fibronectin [14] and expression of integrin receptors in tumor cells [6], [7], which facilitate cell adhesion and the cell-to-cell interaction of tumor cells with the extracellular matrix of BM-derived stromal cells [15]. In turn, hematopoietic progenitors are capable of producing and responding to TGF-β1, and the effects of autocrine TGF-β1 signaling have been shown to induce HSC cell quiescence [9], [10]. Furthermore, TGF-β1 can induce expression of the chemokine receptor CXCR4 through activation of Smad2/3 [17], [18]. CXCR4 is highly expressed in AML, and interactions between CXCR4 and its ligand CXCL12, constitutively secreted by BM stromal cells, promote proliferation, survival, migration, and homing of cancer cells [16]. In this context, we proposed that abundant TGF-β within the BM niche may play an essential role modulating sensitivity of acute myeloid leukemia (AML) cells to chemotherapeutic agents.

Recent data indicate that hypoxia, present primarily along endosteum at the bone-BM interface, is an integral feature of the normal and leukemic bone marrow microenvironment [19], [20]. We have recently shown that progression of leukemia is associated with vast expansion of the bone marrow hypoxic areas and that hypoxia contributes to chemoresistance of leukemic cells [21].

In several systems, hypoxia appears to activate TGF-β signaling, for example by increasing *TβR-I* and *TβR-II* mRNA levels in human fibroblasts [22], or by stimulation of phosphorylation, nuclear transport and transcriptional activities of Smad2 and Smad3 proteins in human umbilical vein endothelial cells [23]. Hypoxia-Inducible Factor α (HIF-1α), one of the best characterized markers of hypoxia, is a transcription factor that controls a vast array of gene products involved in energy metabolism, angiogenesis, apoptosis, cell cycle, and has become recognized as a strong promoter of tumor growth [24]. TGF-β is one of the direct transcriptional targets of HIF-1α [22], [23]. Furthermore, we have previously demonstrated that hypoxia increases CXCR4 expression, another target of HIF-1α [25], leading to increased migration and survival of leukemic cells [26].

To study the role of TGF-β in AML cell survival under conditions mimicking hypoxic BM microenvironment, we investigated the antileukemic effects and molecular mechanisms of action of monoclonal pan–TGF-β-neutralizing antibody, 1D11 [27]. We further investigated the antileukemic efficacy of 1D11 combined with CXCR4 antagonist Plerixafor in an *in vivo* leukemia model. A receptor tyrosine kinase FMS-like tyrosine kinase-3 (FLT3) [28] is constitutively activated by internal tandem duplications (FLT3/ITD mutations) in approximately 30% of de novo AML patients [29], [30], which is recognized to cause a greater relapse rate and a poorer overall survival in AML patients [29]. We therefore utilized FLT3-mutated leukemia cell lines of MV4;11 and Ba/F3-ITD to confirm the cell protective effects of TGF-β on FLT3-ITD positive cells under BM microenvironment. We also examined the effects of TGF-β on wt-FLT3 AML cell lines and AML primary samples harboring wt-FLT3 as well as FLT3-ITD. Our findings indicate that blockade of TGF-β signaling inhibits important pro-survival signals within tumor microenvironment critical for survival of resident chemoresistant leukemia progenitor cells.

## Materials and Methods

### Cell culture

Human AML cell lines MV4;11, U937 and THP-1 were purchased from the American Tissue Culture Collection (Manassas, VA). MV4-11 cells express FLT3/ITD mutation in homozygous form [31], and U937 and THP-1 cells express wt-FLT3. The Ba/F3-ITD cell line was generated by stably expressing a FLT3/ITD cDNA in the interleukin 3 (IL-3)-dependent mouse pro-B lymphocyte line Ba/F3 [31], [32]. Cells were cultured in RPMI 1640 medium containing 10% heat-inactivated fetal bovine serum (FBS), 1% L-glutamine, 100 U/ml penicillin, and 100 µg/ml streptomycin at 37°C in 5% CO_2_. Growth medium of BaF3 cells was supplemented with IL-3 (1 ng/mL). Primary AML samples were obtained after informed consent in accordance with institutional guidelines set forth by Aichi Medical University per Declaration of Helsinki principles. Mononuclear cells were purified by Ficoll-Hypaque (Sigma-Aldrich, St Louis, MO) density-gradient centrifugation, and nonadherent cell were resuspended in RPMI 1640 medium supplemented with 10% FBS at a density of 6×10^6^ cells/mL. Clinical characteristics of patients are summarized in Table S1.

Mesenchymal stem cells (MSCs) obtained from BM of healthy donors were cultured at a density of 5,000 to 6,000 cells/cm^2^ in minimum essential medium alpha supplemented with 20% FBS, 1% L-glutamine, and 1% penicillin-streptomycin as described elsewhere [33]. The isolated, cultured MSCs at passage 3 comprised a single phenotypic population, as determined by flow cytometric analysis, positive for SH2 and SH3 and negative for markers of hematopoietic lineage, as described elsewhere [34]. Passage 3 or 4 MSCs were used for the co-culture experiments. To study the effect of BM stroma on AML cells, AML cell lines or primary AML cells were cultured, at a density of 1×10^5^ or 1×10^6^, respectively, with or without a layer of MSCs plated at a density of 0.2×10^5^ cells/cm^2^. Co-cultured AML cells were separated from the MSC monolayer by careful pipetting with ice-cold phosphate-buffered saline solution (PBS), repeated twice. After the AML cells were collected, to rule out the possibility of contamination with MSCs, MSC monolayers were examined by microscopy (×100) to confirm that the monolayer was not damaged and that fewer than 10 leukemic cells/visual field remained attached. To verify lack of significant contamination in collected leukemic cells, the expression of CD45 was measured by flow cytometry as a discriminator between leukemic cells and MSCs [32]. Standard laboratory (normoxic) conditions comprised 21% O_2_ and 5% CO_2_ at 37°C. For experiments in a reduced oxygen environment, cells were incubated in 1% O_2_ and 5% CO_2_ at 37°C. Cells were incubated for 3–7 days before experiments to allow adjustment to hypoxic conditions.

### Reagents

Human recombinant TGF-β1 (rhTG-Fβ1) was purchased from R&D Systems (Minneapolis, MN), cytarabine (Ara-C) was obtained from Nippon Shinyaku (Kyoko, Japan). Reagents were diluted to the indicated concentrations with culture medium prior to *in vitro* exposure of cells. A murine antibody that neutralizes all three major active TGF-β isoforms, 1D11 [27], and the isotype-matched murine IgG1 monoclonal control antibody directed against Shigella toxin, 13C4, were provided by Genzyme (Framingham, MA). CXCR4 inhibitor plerixifor was also provided by Genzyme.

### Cell viability and apoptosis analysis

Cell proliferation and viability was determined using the trypan blue dye exclusion method or the CellTiter 96 AQueous One Solution Cell Proliferation Assay (MTS, Promega, Madison, WI) following the company's protocol. Apoptotic cell death was evaluated through annexin V (Roche Diagnostic, Indianapolis, IN) and propidium iodide (PI) staining by a FACScan flow cytometer and Cell Quest software (Becton Dickinson Immunocytometry Systems, San Jose, CA) [33].

### Flow cytometry analysis of cell cycle

Cell cycle distribution was determined by flow cytometric analysis of PI-stained nuclei. DNA content was determined by FACScan flow cytometer and CellQuest software. Briefly, cells were washed twice with PBS, fixed in ice-cold ethanol (70% vol/vol in water), and stained with the PI solution (25 µg/ml PI, 180 U/ml RNase, 0.15% Triton X-100, and 30 mg/ml polyethylene glycol in 4 mmol/l citrate buffer, pH 7.8; all from Sigma-Aldrich, St. Louis, MO) overnight at 4°C.

### Flow cytometry analysis of CXCR4 expression

For CXCR4 expression studies, cells were washed with a 20-fold volume of ice-cold buffer without FBS, stained at 4°C with saturating concentrations of phycoerythrin-conjugated anti-CXCR4 monoclonal antibody (12G5; DAKO Cytomation), and then analyzed by flow cytometry.

### Western blot analysis

For collection of total cell lysates, cells were subjected to solubilization in lysis buffer (PBS, 1× cell lysis buffer [Cell Signaling Technology, Beverly, MA], 1× protease inhibitor [Roche], and 1× phosphatase inhibitor cocktails I and II [EMD Biosciences, San Diego, CA]) and incubated for 30 minutes on ice. The lysates were then subjected to centrifugation for 10 minutes at 13,000 rpm at 4°C, and the supernatants were further analyzed. Protein concentrations were determined by the Bio-Rad Protein Assay Kit (Bio-Rad Laboratories, Hercules, CA) according to the manufacturer's protocol. Total protein (20 µg) was separated by sodium dodecyl sulfate–polyacrylamide gel electrophoresis (Bio-Rad Laboratories) and transferred to polyvinylidene-fluoride membranes (0.45 µm, GE Healthcare, Buckinghamshire, UK), then probed with primary and secondary antibodies according to the manufacturers' protocols. Each membrane was probed for α-tubulin (Sigma-Aldrich) as loading control after being stripped with stripping buffer (Pierce Chemical Co., Rockford, IL). Proteins were visualized by the ECL Plus Western Blotting Detection System kit (GE Healthcare), detected by a luminescent image analyzer (LAS-100 plus; Fujifilm, Tokyo, Japan), and quantified by Image Gauge (Fujifilm).

For immunoblotting, the following antibodies were used: α-tubulin (Sigma-Aldrich), p21 (BD-Pharmingen, San Diego, CA), phosphorylated (p-) Smad2 and horseradish peroxidase–linked anti-mouse and anti-rabbit IgG, both from Cell Signaling Technology.

### Chemotactic assay

For CXCL12 chemotaxis assays, cells (0.5×10^6^) were pretreated with indicated reagents in 10% FBS-containing RPMI medium for 16 hours, then cells were stimulated with CXCL12 (100 ng/mL) for 4 hours at 37°C. The CytoSelect cell migration assay kit (Cell Biolabs, Inc., San Diego, CA) was utilized. The chemotactic index was determined as follows: (number of cells migrating to CXCL12)/(number of cells migrating to medium alone).

### Ba/F3-ITD/luc/GFP murine tumor model

All animal work was done in accordance with a protocol approved by the Institutional Animal Care and Use Committee of The University of Texas MD Anderson Cancer Center. C.B-17 SCID (4- to 6-week-old) female mice (Harlan Sprague-Dawley, Madison, WI) were injectiedi.v. with Ba/F3-ITD/*luc*/GFP cells (0.5×10^6^). Starting on day 3 after Ba/F3-ITD/*luc*/GFP cell injection, mice (seven mice per group) were treated as follows: with Ara-C alone (50 mg/kg, by intraperitoneal injection, weekly), with 1D11 alone (0.5 mg/kg, by intraperitoneal injection, every other day), with AMD3100 alone (10 mg/kg, by subcutaneous injection, every other day), or with a combination of these agents.

Tumor infiltration was monitored by bioluminescence imaging as described elsewhere [35]. Briefly, animals were noninvasively imaged using the In Vivo Imaging System (IVIS-200; Xenogen, Hopkinton, MA) after injection with the luciferase substrate colenterazine (native; Biotium, Hayward, CA). Total body bioluminescence was quantified in a region of interest drawn around each mouse.

The extent of leukemic infiltration of different organs was assessed by hematoxylin and eosin (H & E) staining or anti-GFP staining on day 17. Overall survival of the mice in each group was estimated by the Kaplan-Meier method.

### Immunohistochemistry

Mouse organs were harvested and fixed by immersion in 4% paraformaldehyde. Sections (11 µM) were stained with H & E (×5000; Sigma-Aldrich) and analyzed by light microscopy. For GFP staining, the tissue sections were incubated for 1 hour in blocking solution (1× PBS, 0.5% Tween-20, 0.1% bovine serum albumin) and 10% FBS and then incubated overnight with the anti-GFP antibody (Santa Cruz Biotechnology, Santa Cruz, CA). After washing, sections were incubated for 1 hour with secondary antibody and washed in PBS three times, and coverslips were mounted with Fluoromount-G (Electron Microscope Science, Hatfield, PA). The slides were analyzed under a 60×/1.40 PlanApo objective lens on an Olympus FV500 confocal microscope with Fluoview version 4.3 software (Olympus, Melville, NY).

### Statistical analysis

Results are shown as the mean plus or minus the SD or SEM of the results of at least three experiments. The Student paired *t*-test was used for statistical comparison between groups. *P*-values less than 0.05 were considered statistically significant.

## Results

### TGF-β protects AML cells from Ara-C induced apoptosis

**Figure 1:**
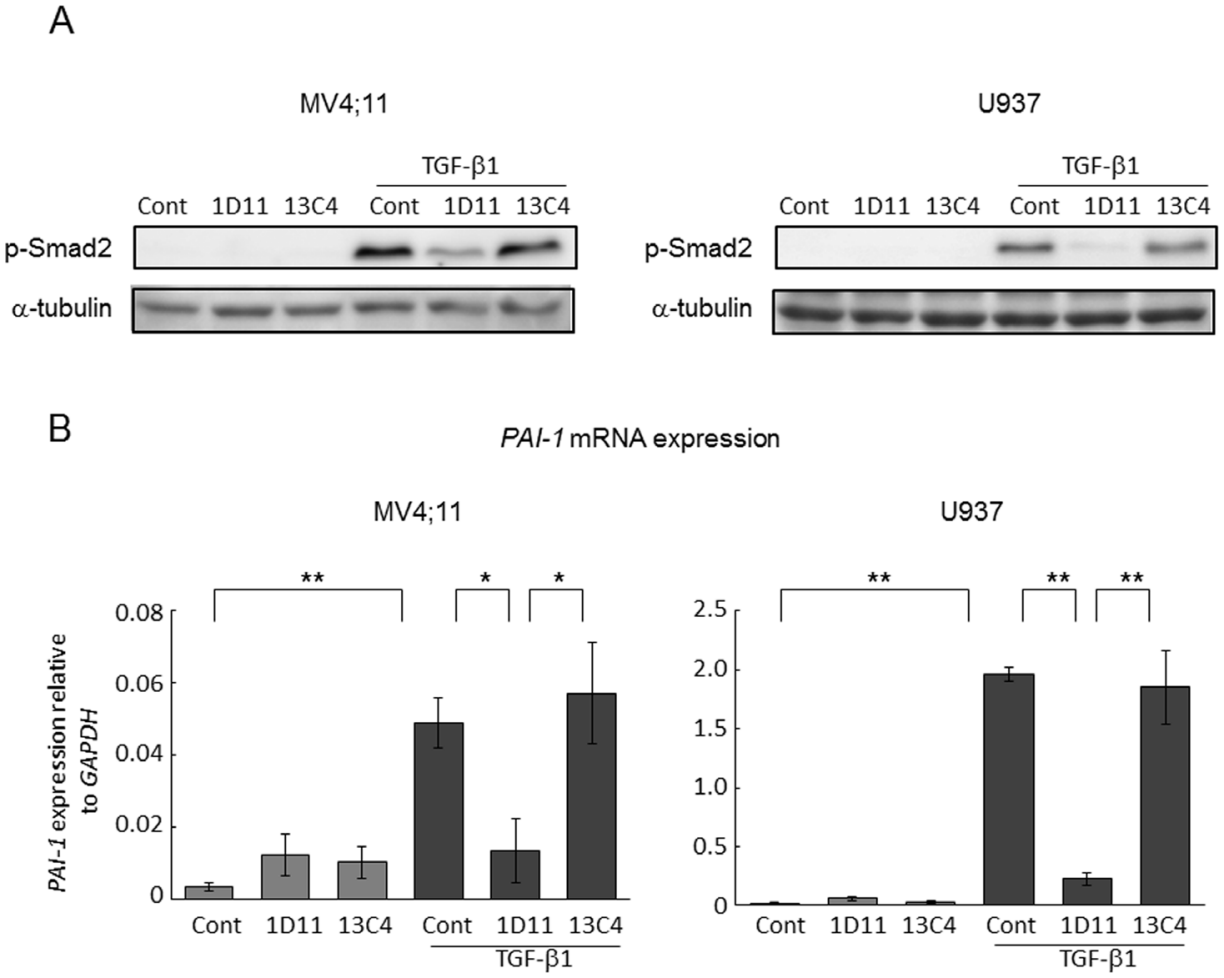
TGF-β-neutralizing antibody1D11 suppresses TGF-β signaling in AML cells. (A) Percentage of MV4;11, U937 and THP-1 cells showing annexin V positivity after 72 hours of treatment with or without rhTGF-β1 (2 ng/ml) and the indicated concentrations of Ara-C. Graphs show the means ± SD of the results from three independent experiments. (B)(C) MV4;11 and U937 cells were treated with TGF-β1 (2 ng/ml), 1D11 (10 µM), or 13C4 (10 µM) for 24 hours. (B) Expression of phospho (p-) Smad2 and p21 proteins by western blot analysis. Clarified lysates were probed with antibodies to p-Smad2, p21 and α-tubulin. Results shown are representative of three independent experiments. (C) *PAI-1* (plasminogen activator inhibitor-1) mRNA expression detected by TaqMan RT-PCR analysis. The abundance of transcripts of *PAI-1* relative to the abundance of transcripts of *GAPDH* was determined as described in Materials and Methods. Results shown are representative of three experiments. *P<0.05, ** *P*<0.01.

In normal haematopoiesis, TGF-β regulates quiescence and survival of HSC, yet its role in leukemic BM microenvironment is not well defined. TGF-β is one of the major factors secreted by BM stromal cells, and we previously reported that TGF-β affects AML cell survival in cell type-specific manner [11]. Whereas Ara-C is a highly effective chemotherapeuric agent for AML therapy, stromal cells protect leukemic cells from Ara-C-induced apoptosis in BM microenvironment [11], [36]. We first examined effects of rhTGF-β1 on apoptosis induced by Ara-C in AML cells. Treatment with 2 ng/mL rhTGF-β1, equivalent to the levels of TGF-β produced by osteoblastic cell lines [37], reduced apoptosis induction by Ara-C in both FLT3/ITD mutant MV4;11 and in wt-FLT3 U937 and THP-1 AML cells (Figure 1A). These data are consistent with our published findings and support the pro-survival role of TGF-β within BM microenvironment, and provide basis for the exploration of therapeutic blockade of TGF-β in AML.

### Suppression of TGFβ signaling by neutralizing antibody 1D11 in AML cells

1D11 is a humanized form of monoclonal antibody that neutralizes 3 major active TGF-β isoforms (TGF-β1, -2, and -3), and does not bind other ligands in the TGF-β superfamily, including multi-functional growth factors activin or bone morphogenitic proteins (BMPs) [27]. To determine the efficacy of TGF-β specific inhibition, we investigated the effects of TGF-β neutralizing antibody 1D11 on exogenous TGF-β induced Smad2 phosphorylation, a first required step in TGF-β signaling, and transcription of TGF-β target gene *PAI-1* in AML cells. MV4;11 and U937 cells were treated with 10 µM 1D11 or isotype control antibody 13C4 in the presence of absence of 2 ng/mL rhTGF-β1. Treatment with rhTGF-β1 induced phosphorylation of Smad2 in both cell lines as detected by western blot (Figure 1B). Real-time reverse-transcription (RT)-PCR demonstrated that rhTGF-β1 induced TGF-β target gene *PAI-1* expression ( Figure 1C ), indicating that TGF-β signaling is intact in these AML cells. Of importance, 1D11 but not control antibody 13C4 fully blocked TGF-β signaling signaling exemplified by these molecular markers. Consistent with established role of TGF-β in quiescence of hematopoietic cells, rhTGF-β1 induced upregulation of cell cycle inhibitor p21 in AML cells, which was abrogated by 1D11 but not by control antibody 13C4 ( Figure 1B ).

### 1D11 reverses TGF-β1-induced cell cycle arrest and apoptosis in AML cells under normoxic and hypoxic conditions

**Figure 2:**
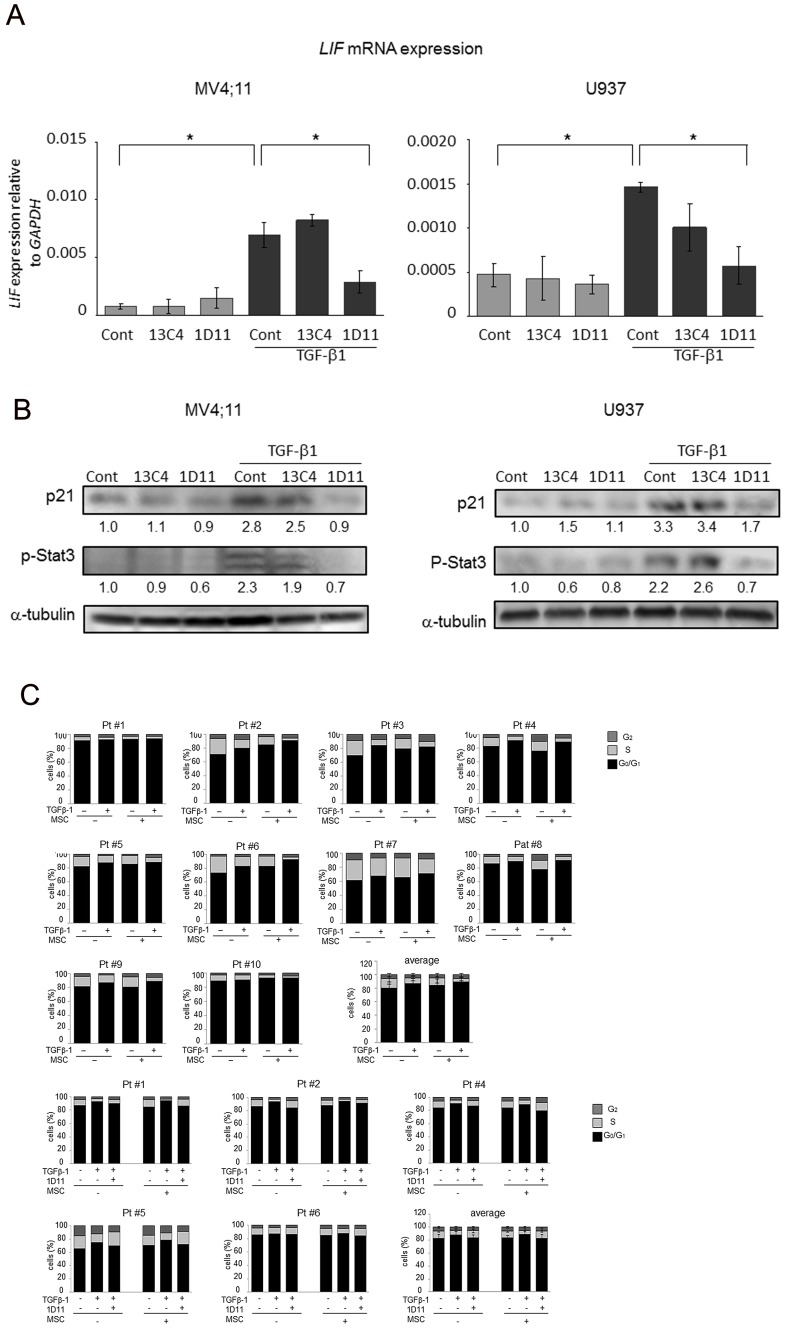
1D11 reverses TGF-β-mediated cell cycle inhibition and anti-apoptotic effects. (A)(B) MV4;11 cells were treated with rhTGF-β1 (2 ng/ml), with and without 1D11 (10 µM) or 13C4 (10 µM), for 72 hours under serum-starved conditions and cultured without and with MSCs, as described in Materials and Methods. (A) The percentage of Annexin V–positivity by flow cytometry analysis. Bar graph represent percentages of annexin V–positive cells (mean± SD) from three independent experiments. (B) Flow cytometric cell cycle analysis by PI staining. Bar graphs represent percentages of SubG_1_- and G_0_/G_1_-phase cells (mean± SD) from three independent experiments. Graphs show the means ± SD of the results from three independent experiments. *P<0.05, **P<0.01. (C) Primary AML cells harboring mutant FLT3-ITD (Pt #1–3) or wt-FLT3 (Pt #4–10) were incubated for 48 h with rhTGF-β1 (2 ng/ml), 1D11 (10 µM), with or without MSCs co-culture condition. Clinical characteristics of patients are summarized in Table S1. The percentage of G_0_/G_1_-, S- and G_2_/M-phase cells were detected by flow cytometric PI cell cycle analysis. Average show the means ± SEM of the results from ten (i) or six (ii) primary AML cells, respectively.

We next examined whether 1D11 could affect pro-survival functions of rhTGF-β1, including conditions of co-culture with BM-derived stromal cells (MSC). Whereas we previously shown that MSCs secret 0.6–1.0 ng/mL of TGF-β1 [11], the levels of TGF-β are higher within the endosteal BM microenvironmental niche [3]. Osteoclastic bone resorption produces abundant active TGF-β from bone, the largest latent reservoir of TGF-β [3]. Therefore, we tested the effects of 1D11 under MSC co-culture conditions supplemented with rhTGF-β1 to mimic the high level of TGF-β concentration in BM microenvironmental niches. We first confirmed the significant protective effects of MSC on the survival of MV4;11 cells upon serum withdrawal (*P*<0.001). In the absence of MSC, rhTGF-β diminished spontaneous apoptosis of MV4;11 cells, and this effect was largely reversed by 1D11. In a co-culture setting, rhTGF-β1 and 1D11 only slightly modulated pro-survival effects of MSC, indicating that cell-to-cell interactions of tumor cells with the extracellular matrix, or growth factors secreted by MSC provide redundancy in the anti-apoptotic mechanisms of protection (Figure 2A , Figure S1A) [2], [15].

TGF-β is a known mediator of cell cycle quiescence in normal hematopoietic progenitor cells [38]. In our system, rhTGF-β1 increased the proportion of cells in G_0_/G_1_ phase and decreased the fraction of proliferating (S-phase) MV4;11 cells ( Figure 2B , Figure S1B). Importantly, the proportion of cells in G0/G1 cell cycle phase upon rhTGF-β1 exposure was further increased in the presence of MSCs (53.6±3.5% vs. 79.5±4.0%, p<0.001). 1D11 abrogated rhTGF-β1 induced cell cycle arrest even when AML cells were co-cultured with MSC. The control antibody 13C4 affected neither pro-survival effects nor cell cycle arrest mediated by rhTGF-β1 ( Figure 2 , Figure S1). Analysis of sub-G1 fraction confirmed findings by annexin V flow cytometry, in that TGFβ or MSC significantly inhibited serum-withdrawal-induced cell death, and 1D11 abrogated effects of TGFβ but not of MSC ( Figure 2 , Figure S1). These findings suggest that TGFβ and MSC exert pro-survival and anti-proliferative effects through different mechanisms. We next tested effects of rhTGF-β1under MSC co-culture conditions in primary AML samples. Because mutation in FLT3 gene is one of the most common driver mutations in AML and confers poor prognosis, we utilized primary AML cells with or without FLT3-ITD mutation (see Table S1 for further details on clinical characteristics). We observed moderate increase in the proportion of the cells in G0/G1 cell cycle phase after rhTGF-β1 exposure in all primary AML samples, which was more prominent in the presence of MSCs irrespective of FLT3 status (8/10 cases; Pt #1,2,5–10). The cell cycle arrest induced by rhTGFβ1 was abrogated by 1D11 when cultured alone or in the presence of MSCs in all tested primary AML samples (Pt #1,2,4–6) (Figure 2C).

These results suggest that high levels of TGF-β1 contribute to the accumulation of quiescent AML cells within BM microenvironment, and these effects are reversed by TGF-β neutralizing antibody 1D11.

### Combination of 1D11 and Ara-C induces apoptosis in AML cells

**Figure 3:**
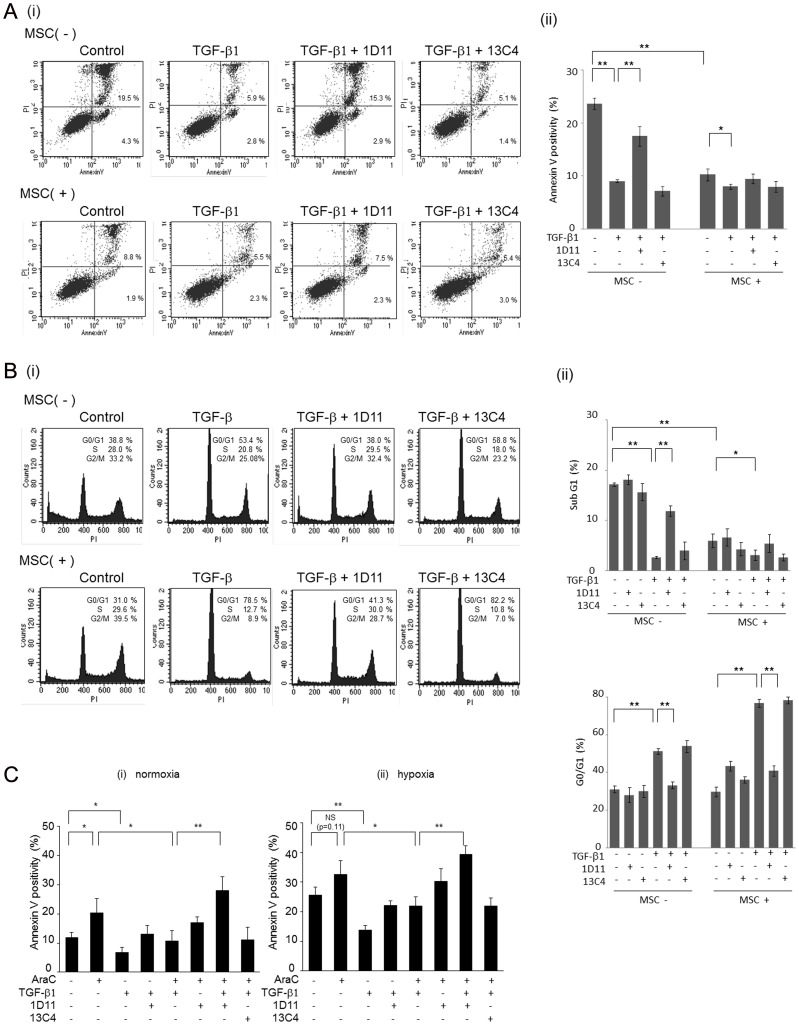
1D11 enhances the proapoptotic effect of Ara-C in both normoxic and hypoxic conditions. MV4;11 cells were treated with Ara-C (0.5 µM), rhTGF-β1 (2 ng/ml), 1D11 (10 µM), and/or 13C4 (10 µM) for 72 hours under normoxic (A) and hypoxic (B) conditions. Percentages of apoptotic cells (annexin V–positive cells) were analyzed by flow cytometry. Graphs show the means ± SD of the results from three independent experiments. *P<0.05, **P<0.01.

Hypoxia is an essential component of BM microenvironmental niches, and we have demonstrated the contribution of the hypoxia to chemoresistance of leukemic cells [21]. HIF-1α, a well characterized marker of hypoxia, upregulates its direct transcriptional target TGF-β and functions as a critical mediator of TGF-β signaling in hypoxic condition [22], [23]. To investigate if 1D11 efficiently abrogates the TGF-β1 mediated prosurvival effect on AML cells under hypoxic condition, Ara-C exposed MV4;11 cells were subjected to the treatment with rhTGF-β1 with/without 1D11 or 13C4 under normoxic and hypoxic conditions. As shown in Figure 3, rhTGF-β1 reduced spontaneous and Ara-C-induced apoptosis under hypoxia with a similar trend for normoxic conditions. While 1D11 had no apoptotic effect on its own, combination of 1D11 with Ara-C reversed protective effects of TGF-β1 and induced higher degree of apoptotic cell death than Ara-C alone. Treatment with 1D11 combined with Ara-C without rhTGF-β1 showed no significant effect. This suggests that 1D11 effectively abrogates TGF-β-mediated chemoresistance of AML cells acquired in hypoxic BM microenvironment.

### Effects of combined blockade of CXCR4 and TGF-β pathways in AML cells

Prevention of AML cell migration to BM microenvironment along with blockade of cell-to-cell interaction with BM stromal cells is an important strategy thought to abrogate microenvironment-mediated chemoresistance. Migration of AML cells is regulated by stromal cells producing CXCL12 [16]. Recently, several studies have demonstrated the positive transcriptional regulation of CXCR4 by Smad2/3, which highlights the direct association between TGF-β and CXCL12/CXCR4 signaling [17], [18]. We therefore examined simultaneous blockade of CXCL12/CXCR4 and TGF-β signaling on AML cell survival in BM microenvironment.

**Figure 4:**
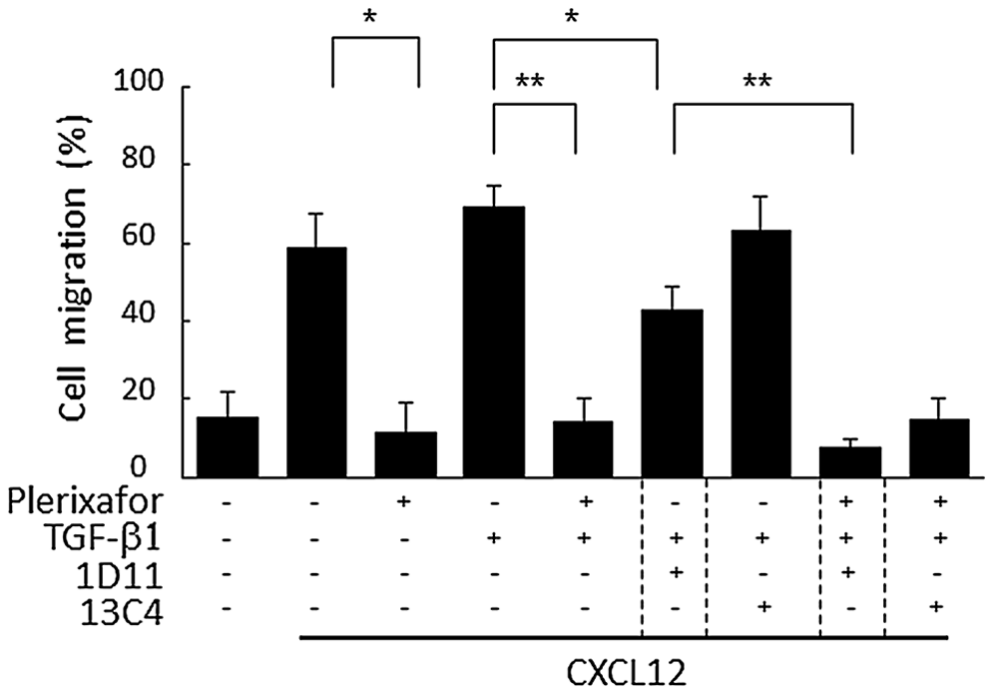
Plerixafor and 1D11 inhibit CXCL12-induced migration. Chemotaxis assay of MV4;11 cells treated with TGF-β1 (2 ng/ml), 1D11 (10 µM), and/or plerixafor (1 µM) for 18 hours. Cells were stimulated with CXCL12 (100 ng/mL) for 4 hours. In all experiments, the chemotactic index was calculated as described in Materials and Methods. Graphs show the means ± SD of the results from three independent experiments. *P<0.05, **P<0.01.

First, we evaluated the effect of combined blockade of CXCL12/CXCR4 and TGF-β pathways on CXCL12-induced migration. A receptor antagonist Plerixafor is known to block active CXCL12/CXCR4 signaling in normal and leukemia stem cell [39]. As shown in Figure 4, Plerixafor completely inhibited MV4;11 cells migration to CXCL12 irrespective of presence or absence of rhTGF-β1 and/or 1D11. Whereas rhTGF-β1 alone did not cause cells to migrate and did not further enhance CXCL12-triggered cell migration, blockade of TGF-β by 1D11 partially but significantly inhibited cell migration to CXCL12 in the presence of rhTGF-β1, suggesting positive regulation of cell migration by active TGF-β signaling. No significant increase in CXCR4 expression was observed in cells exposed to rhTGF-β1 (data not shown). Plerixafor, alone or combined with 1D11, did not affect rhTGFβ1-mediated reduction in spontaneous apoptosis or cell cycle arrest in MV4;11 cells co-cultured with MSC *in vitro* (data not shown).

### Combination of 1D11/plerixafor/Ara-C induced potent antitumor effects *in vivo*

**Figure 5:**
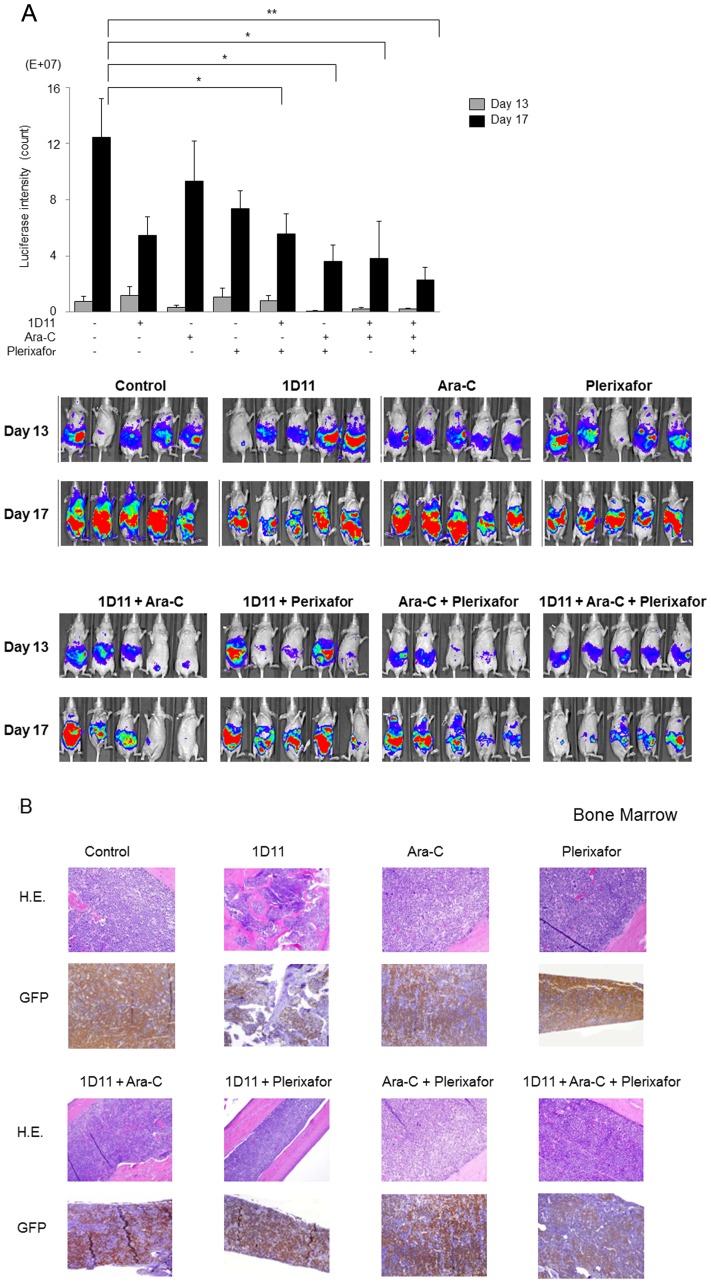
Combination of 1D11, Plerixafor, and Ara-C induces potent antitumor effects ***in vivo*** **.** Ba/F3-ITD-luciferase leukemia cells were injected into nude mice as described in Materials and Methods. (A) Serial bioluminescence images (i) and luciferase intensity (ii) of mice in the groups receiving 1D11, Ara-C, Plerixafor, one of the combinations indicated, or no treatment (control) were taken on days 13 and 17 after tumor cell injection. *P≤0.05, **P<0.01. (B) Histologic sections of bone marrow taken from mice on day 17 were stained with H&E or anti-GFP antibody.

Taken into consideration limitations of the in vitro systems in the assessment of agents affecting tumor microenvironment, we evaluated the utility of combined blockade of CXCL12/CXCR4 and TGF-β signaling pathways in the in vivo leukemia model. To this end, we utilized a murine model of FLT3-mutated AML by transplanting mice with Ba/F3-ITD-luciferase leukemia cells known to engraft primarily in the BM of mice with secondary dissemination to the spleen and other organs [32]. Mice were treated with1D11, Ara-C, or plerixafor, alone or in combinations. Bioluminescence imaging on day 17 showed that while Ara-C alone did not significantly impact tumor growth, 1D11 combined with Ara-C or with Plerixafor significantly decreased leukemia burden (*P* = 0.05). Plerixafor combined with Ara-C also showed anti-leukemia effect consistent with our published data [40]. In turn, co-administration of all three agents together most potently decreased leukemia burden (*P* = 0.003) ( Figure 5A ). This was confirmed by significant decrease in the proportion of GFP-positive leukemia cells in the BM (Figure 5B) and spleen (not shown) of mice co-treated with 1D11, Ara-C, and plerixafor. Despite this transient anti-tumor response, all mice succumb from leukemia in this very aggressive AML model, whereby three-agent combination only modestly extended survival (*P* = 0.015, Table S2).

## Discussion

**Figure 6:**
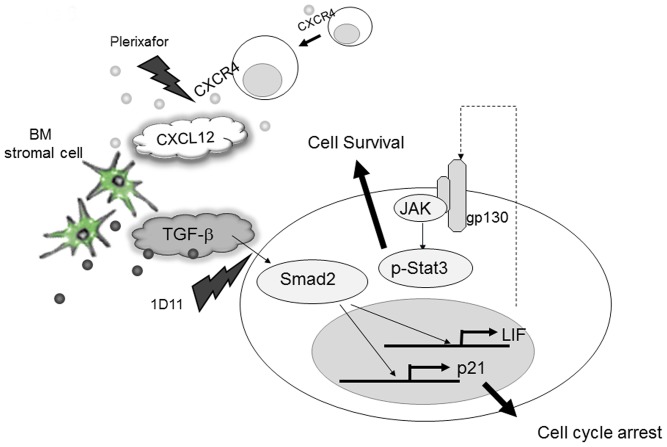
Simultaneous blockade of TGF-β and CXCL12/CXCR4 signaling may enhance the efficacy of chemotherapy against AML cells in the hypoxic BM microenvironment. Within the BM microenvironment, TGF-β secreted by stromal cells induces cell cycle arrest of AML cells through p21 upregulation that is inhibited by 1D11. Combination of Plerixafor, which inhibits CXCL12-induced migration, and 1D11, which blocks TGF-β signaling, abrogates stroma-mediated chemoresistance and promotes the antileukemia effects of chemotherapy.

TGF-β is known as one of the key regulators of tumor-stroma interactions. In this study, we dissected the functional role of TGF-β within leukemic BM microenvironment and identified the pro-survival effects of TGF-β in AML cells. TGF-β has been hypothesized to be a cardinal regulator of the natural quiescence of hematopoietic stem cells, maintaining these cells in a slow-cycling state *in vivo* [9], [10], [41]. Several molecular mechanisms have been proposed to account for TGF-β-mediated growth inhibition, including alterations in cytokine receptor expression and upregulation of cyclin-dependent kinase inhibitors such as p15, p21, and p27 [42]. Similar to its effects in normal hematopoietic progenitors, rhTGF-β1 induced p21 upregulation ( Figure 6 ) and accumulation of AML cells in a quiescent G_0_ state, and this accumulation was prevented by TGF-β-neutralizing antibody 1D11. Remarkably, the proportion of cells in G0/G1 cell cycle phase was further increased under MSC co-cultures in both, AML cell lines and in primary AML samples, suggesting that additional microenvironmental cues regulate cell cycle state of leukemic cells. Cell cycle quiescence is a known mechanism of stroma-induced resistance against cell cycle-specific chemotherapy agents, and based on our findings we propose that this effect is mediated at least in part via paracrine TGF-β production.

Very recently, disruption of the normal bone marrow microenvironment through leukemia-driven inhibition of osteoblastic function and transient increase of osteoclasts has been demonstrated in a murine model of AML [43]. Bone is the largest reservoir of TGF-β, a key regulator of mechanical properties of the bone matrix [44], which can modulate the functions of MSCs in the bone microenvironment. Upon reaching the bone microenvironmental niches, AML cells are exposed to a high level of active TGF-β, which in turn mediates a cascade of events that favor the tumor cell survival. We therefore further investigated the role of TGF-β under conditions mimicking BM microenvironment. Using *in vitro* co-culture assay system, we have demonstrated that 1D11 abrogated cell cycle arrest by the excess TGF-β1. These effects were seen in AML samples regardless of their FLT3 mutation status, indicating that microenvironmental regulation is broadly applicable to diverse AML subsets. Notably, 1D11 enhanced Ara-C–induced apoptosis in the MV4;11 cells not only under normoxic but also under hypoxic conditions prevalent in leukemic BM microenvironment. These findings suggest prominent role of TGF-β in AML cell survival under hypoxia. HIF-1α also functions as a transcriptional activator of CXCR4 [25], and we demonstrated that CXCL12/CXCR4 is an important component of the hypoxic microenvironment [26]. Several studies have indicated that TGF-β emits protumorigenic signals to surrounding cells through CXCL12/CXCR4 signaling pathways [45], [46]. In our study, TGF-β blockade by 1D11 significantly inhibited AML cell migration in the presence of exogenous TGF-β1. Recently, autocrine signaling loops of TGF-β and CXCL12 have been reported in myofibroblasts, whereas active TβR-Smad2/3 increases SDF-1α expression, thereby boosting CXCL12/CXCR4 autocrine signaling [47]. It is possible that abundant exogenous TGF-β may trigger autocrine production of CXCL12 by AML cells and further stimulate CXCL12/CXCR4 signaling, which is diminished by 1D11. The additional effects of 1D11 and Plerixafor combination observed in the *in vivo* model might be further explained by the different mechanisms of action of these two reagents; Plerixafor is a potent inhibitor of leukemia cell migration but does not affect cell cycle progression, while 1D11 has moderate effects on migration but potently inhibits G0/G1 cell cycle arrest. These interactions appear to play a prominent role in vivo, pointing to the limitations of the in vitro co-culture systems and to the importance of utilization of the murine leukemia models for testing agents affecting leukemia/microenvironment interactions. Consistent with the notion of a functional cross-talk between TGF-β and CXCR4 pathways under conditions of leukemic BM microenvironment, combination of 1D11 and Plerixafor demonstrated a remarkable ability to enhance antileukemic effect of Ara-C in the in vivo mouse model.

In summary, these data demonstrate that TGF-β signaling plays an important role in microenvironment-mediated chemoresistance in AML, and that TGF-β blockade in conditions of interacting hypoxia and BM stromal cells may be an effective new tool in the therapy of genetically diverse AML subsets. Of note, 1D11 has shown no overt toxicities in healthy animals [48], and currently, the human equivalent of 1D11, designated as GC1008, is undergoing phase I clinical trials in human cancer patients [49].Our findings thus argue for rationale combinations of agents targeting leukemic BM microenvironment and conventional cytotoxic drugs with the goal to overcome stroma-mediated chemoresistance in AML.

## Supporting Information

Figure S1 **1D11 reverses TGF-β-mediated cell cycle inhibition and anti-apoptotic effects.** MV4;11 cells were treated with rhTGF-β1 (2 ng/ml), with and without 1D11 (10 µM) or 13C4 (10 µM), for 72 hours under serum-starved conditions and cultured without and with MSCs, as described in Materials and Methods. Graphical representations of FACS data with representative percentages of Annexin V–positivity (A) and of G_0_/G_1_-, S- and G_2_/M-phase cells detected by PI staining (B).
(DOCX)

Table S1 **Clinical characteristics of AML patients.**
(DOCX)

Table S2 **Survival of 1D11, Ara-C and Plerixafor treated mice.**
(DOCX)
