# Supplementary material for: TGF-β-Neutralizing Antibody 1D11 Enhances Cytarabine-Induced Apoptosis in AML Cells in the Bone Marrow Microenvironment
Source: PLoS One. 2013 Jun 27;8(6):e62785. doi: 10.1371/journal.pone.0062785 (PMC3695026; doi:10.1371/journal.pone.0062785)

**Supplementary Figure S1. TGF-β1 inhibits Ara-C−induced cell death in AML cells**. Percentage of U937, MV4;11 and THP-1 cells showing annexin V positivity (S1A) and viable cell number (S1B) after 72 hours of treatment with or without rhTGF-β1 (2 ng/ml) and the indicated concentrations of Ara-C. Graphs show the means ± SD of the results from three independent experiments. **P* < 0.05.


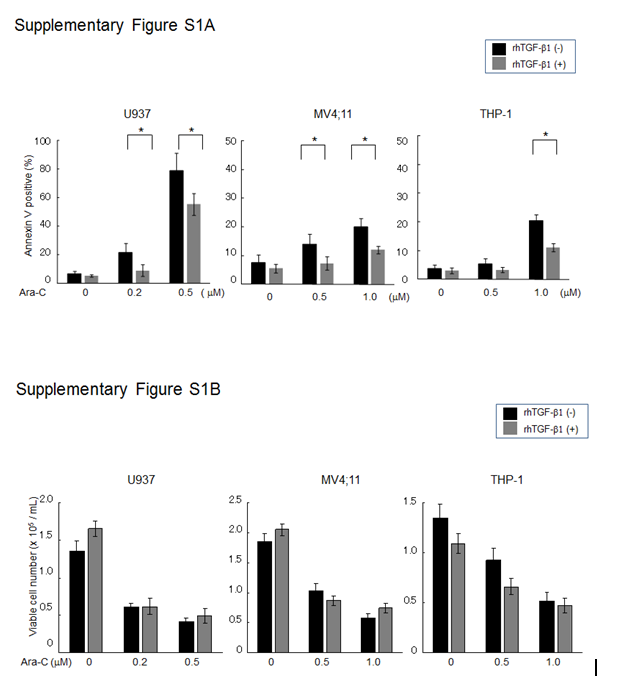

Supplement: Figure S1 — 1D11 reverses TGF-β-mediated cell cycle inhibition and anti-apoptotic effects. MV4;11 cells were treated with rhTGF-β1 (2 ng/ml), with and without 1D11 (10 µM) or 13C4 (10 µM), for 72 hours under serum-starved conditions and cultured without and with MSCs, as described in Materials and Methods. Graphical representations of FACS data with representative percentages of Annexin V–positivity (A) and of G0/G1-, S- and G2/M-phase cells detected by PI staining (B). (DOCX) [file pone.0062785.s001.docx]
