# Supplementary material for: TGF-β-Neutralizing Antibody 1D11 Enhances Cytarabine-Induced Apoptosis in AML Cells in the Bone Marrow Microenvironment
Source: PLoS One. 2013 Jun 27;8(6):e62785. doi: 10.1371/journal.pone.0062785 (PMC3695026; doi:10.1371/journal.pone.0062785)
Supplement: Table S1 — Clinical characteristics of AML patients. (DOCX) [file pone.0062785.s002.docx]

**Supplementary Table S1. Clinical characteristics of AML patients**

| Patient number | source | age/  sex | FLT3 status | diagnosis | WBC  (x10^9^/L) | %  BM Blast  (PB Blast) | Disease status | Cytogenetics |
| --- | --- | --- | --- | --- | --- | --- | --- | --- |
| 1 | PB | 80/F | FLT3-ITD | M4 | 48.9 | 84.4(49) | Primary refractory | 46,XX (20/20) |
| 2 | BM | 63/M | FLT3-ITD | M5 | 265.0 | 79.2(73) | Primary refractory | 46,XY(20/20) |
| 3 | BM | 70/M | FLT3-ITD | M5 | 88.4 | 22.8/52.8*(21) | Primary refractory | 46,XY,del(5)(q?),del(15)(q?) (8/20) 46,sl,t(7;20)(q32;q13.1) (12/20) |
| 4 | BM | 80/M | wt-FLT3 | MDS/AML | 4.7 | 15/83.2**(23) | Primary refractory | 43,XY,add(1)(p11),der(1;4)(p10;p10),del(5),　-7,add(7)(q32),dup(8)(q11.2q24),-9, add(12)(p11.2),-13,16,del(20)(q11.2q13.1), +mar1[2]/43,idem,add(11)(p15)[5]/44,idem, add(12),+mar2,+mar3[6]/46,XY[4] |
| 5 | BM | 79/M | wt-FLT3 | MDS/AML | 16.7 | 32.6(42) | Primary refractory | 46,XY,del(7)(q?)(20/20) |
| 6 | BM | 30/M | wt-FLT3 | M1 | 175 | 91.4(95.8) | CR | 46,XY(20/20) |
| 7 | BM | 22/M | wt-FLT3 | M5 | 11.4 | 95.4(71) | Relapse / refractory | 46,XY,t(6;11)(q27;q23) (19/20) 47,sl,+mar (1/20) |
| 8 | PB | 71/M | wt-FLT3 | M5 | 22.3 | 35.2/29.2*(25) | unknown | 44,XY,del(5)(q?),der(7)t(7;13)(q22;q12),  -12,der(13)t(12;13)(?;p11)t(7;13)(?;q12), der(17)t(17;20)(p11;?),-20[18]/46,XY[1] |
| 9 | BM | 53/M | wt-FLT3 | M5 | 43.8 | 3.4/67.8*(0) | Relapse / refractory | 46,XY (20/20) |
| 10 | BM | 28/M | wt-FLT3 | M5 | 45.4 | 88.4(1.5) | Primary refractory | 46,XY(20/20) |

*immature monocyte
