# Supplementary material for: TGF-β-Neutralizing Antibody 1D11 Enhances Cytarabine-Induced Apoptosis in AML Cells in the Bone Marrow Microenvironment
Source: PLoS One. 2013 Jun 27;8(6):e62785. doi: 10.1371/journal.pone.0062785 (PMC3695026; doi:10.1371/journal.pone.0062785)
Supplement: Table S2 — Survival of 1D11, Ara-C and Plerixafor treated mice. (DOCX) [file pone.0062785.s003.docx]

**Supplementary Table S2.** Survival of 1D11, Ara-C and Plerixafor treated mice

| treatment | median survival | (range) | p |
| --- | --- | --- | --- |
| Control (6)^*^ | 17 days | (16-18) |  |
| 1D11 (7) | 18 days | (16-21) |  |
| Ara-C (7) | 18 days | (17-19) |  |
| Plerixafor (7) | 17 days | (16-19) |  |
| 1D11 + Ara-C (7) | 18 days | (16-19) |  |
| 1D11 + Plerixafor (6) | 17 days | (16-19) |  |
| Ara-C + Plerixafor (6) | 19 days | (16-21) | 0.028 |
| 1D11 + Ara-C + Plerixafor (6) | 19 days | (17-22) | 0.015 |

^*^( ) number of mice
